# A simulation-based learning experience in augmentative and alternative communication using telepractice: speech pathology students’ confidence and perceptions

**DOI:** 10.1186/s41077-019-0113-x

**Published:** 2019-12-20

**Authors:** Simone Howells, Elizabeth A. Cardell, Monique C. Waite, Andrea Bialocerkowski, Neil Tuttle

**Affiliations:** 1 Menzies Health Institute Queensland, Gold Coast Campus, Griffith University, QLD, Southport, 4222 Australia; 2 School of Allied Health Sciences, Gold Coast Campus, Griffith University, QLD, Southport, 4222 Australia; 3 School of Health and Rehabilitation Sciences, The University of Queensland, Brisbane, Australia

**Keywords:** Simulation, Speech-language pathology, Student training, Augmentative and alternative communication, Telepractice, Clinical education

## Abstract

**Background:**

Simulation, as an activity in speech-language pathology training, can increase opportunities for students to gain required skills and competencies. One area that has received little attention in the simulation literature, yet is a growing area of clinical practice, is alternative and augmentative communication (AAC). Also growing, is the use of telepractice to deliver services. This exploratory study investigated graduate entry speech-language pathology student perceptions of a simulation learning experience working with an adult with complex communication needs via telepractice.

**Methods:**

First year Master of Speech Pathology students completed a 1-day simulation using a videoconferencing delivery platform with an actor portraying an adult client with motor neurone disease requiring AAC. Quantitative and qualitative survey measures were completed pre- and post-simulation to explore students’ confidence, perceived impact on clinical performance, and perceived extent of learning, specifically, their interest, competence, and tension. Further, students’ perceptions about the telepractice system useability were explored. Fifty-two responses were received and analysed using descriptive statistics and content analysis.

**Results:**

Post-simulation, students reported increased confidence and perceived positive impacts on their confidence and clinical skills across communication, assessment, and management domains. They felt better prepared to manage a client with a progressive neurological condition and to make AAC recommendations. For telepractice delivery, technology limitations were identified as impacting its use, including infrastructure (e.g., weak internet connection). In addition, some students reported feeling disconnected from the client.

**Conclusion:**

This study supports the use of simulation in AAC through telepractice as a means of supporting Masters-level speech pathology student learning in this area of practice.

## Background

Speech-language pathology (SLP) preparation programmes are underpinned by the inclusion of work-integrated learning, often referred to as clinical education, as a means of producing work-ready graduates [1]. However, the provision of clinical education is becoming increasingly difficult due to a number of factors [2, 3]. The university sector has experienced changes in recent years, including increased numbers of accredited health programmes and health students requiring clinical placements [2, 4]. Financial constraints have led to changes within the SLP workforce, including staff shortages, increased clinician responsibilities and reduced funding for education and training [1, 5–7]. In combination, these factors have influenced the capacity for universities to provide ample and equitable clinical education experiences for students, creating the possibility that some students may not be able to graduate with sufficient practical experience in core practice areas [5, 8].

In response to these drivers, innovative clinical education models have been explored across health professions including SLP [3, 7]. One such innovation is simulation, used as an adjunct to, or a replacement of, traditional clinical placement experiences. Simulation, as defined by Gaba [9] is “a technique, not a technology, to replace or amplify real experiences with guided experiences, often immersive in nature, that evoke or replicate aspects of the real world in a fully interactive fashion”. Simulation, as a learning activity, therefore, has the potential to offer effective and efficient ways to teach and assess students’ clinical performance and may, in turn, address clinical placement capacity issues [10]. Further, simulation offers many benefits to learners that traditional placements alone may not afford, for example, the ability to develop skills in a safe, controlled learning environment where patient safety is ensured [11], repeated practice to consolidate skill development, manipulation of task difficulty, accommodation of student mistakes, and provision of feedback beyond what is typically feasible in a real patient interaction [12]. Hence, it is possible that simulation can offer learners enhanced and adaptively tailored experiences as well as exposure to experiences that traditional clinical placements, alone, may not offer.

Although simulation has long been used in the education and training of medical, nursing, and even military personnel [13], its use in SLP student training is relatively recent [10], but growing [14, 15]. While it is yet to be ascertained whether clinical learning outcomes in SLP are comparable to traditional clinical placements, early data analyses from the Australian National Simulation Project in Speech Pathology randomised controlled trial suggests non-inferiority [15]. Furthermore, randomised control trials in physiotherapy have concluded that simulated clinical activities can be used to replace up to 25% of clinical placement time without any detrimental effect on student outcomes [16] and emerging research suggests simulation may offer physiotherapy students better preparation for placement than traditional placement alone [17, 18]. Indeed, the American Speech-Language-Hearing Association, in 2016, updated their certification standards to allow 75 h of clinical simulation experiences in graduate SLP programmes [14]. A recent national survey of university programmes’ use of simulation in the USA, in which 44% of educators responded, indicated that 51% were using simulation in clinical education and 48.5% of total respondents agreed with the statement that up to 25% of clinical education could be replaced with simulation [19].

Simulation can be high fidelity and provide a high level of realism for the learner through simulated patients (SPs), human patient simulators, mannequins, task trainer, and virtual reality or can be lower in fidelity, that is, more artificial or when an action is closely mirrored but elements are missing from what might be experienced in real life, for example, part-task trainer [20, 21]. Simulation in SLP has predominantly been high fidelity and used face-to- face-simulated patients (SPs) [10, 20, 22, 23] which are actors or real patients trained to portray the characteristics of a real patient including psychological, emotional, historical, and physical traits [21]. Known specific benefits of using SPs include allowing students to train for a clinical scenario without compromising real patient care and bolstering student confidence and comfort through the awareness that SPs are trained actors [5, 20, 22–26].

The first reported use of SPs in SLP programmes took place in Australia in 1995 when Edwards, Franke, and McGuinness [27] used actors to assist students to develop their clinical reasoning. Closely following this, Syder [28] in the UK used simulated patients to portray a client with a suspected voice disorder to develop student practical skills. More recently, SPs have been used in SLP student training to manage difficult clients [27]; develop communication skills [23]; portray the parent of a child with a speech disorder [29], as well as in the areas of aphasia [27, 30, 31]; and Alzheimer’s dementia [28, 31]. Collectively, these studies have demonstrated positive benefits for students’ learning. However, simulation research using SPs in some specialised areas of clinical practice, such as augmentative and alternative communication (AAC), is sparse [32].

### Augmentative and alternative communication

AAC is a globally recognised [33–35] and key area of SLP clinical practice that provides communication strategies, techniques, and interventions for people with a range of communication difficulties and includes the use of gestures, manual signs, picture communication boards, and/or voice output communication devices [34, 35]. AAC is closely integrated into the area of multimodal communication, which is one of the six practice domains, mandated by Speech Pathology Australia [36], in which graduates must demonstrate entry-level competencies. With clinical placements becoming increasingly difficult to source [2, 4, 20], simulation is one means of developing students’ competencies and, furthermore, may facilitate training programmes in meeting accreditation requirements around multimodal communication. Simulation, when applied in this manner, also ensures equitable and standardised whole-cohort student experiences.

To date, research in AAC simulation has been limited to paediatrics. Thistle and McNaughton [37] explored the impact of SLP students practicing active listening skills, through role play with communication partners who were trained special education professionals acting as a child who used AAC, and concluded the simulation was an effective and efficient means for developing active listening skills. However, with one exception [38], there has been no published data on SLP student AAC skill development through simulation, nor investigation specifically targeting AAC for adult populations. In Australia’s current workforce climate, the introduction of the National Disability Insurance Scheme has seen working with clients and their AAC needs assume a new prominence; hence, training students in AAC management needs to be a priority. Simulation learning activities in AAC is one approach that can offer students consistent and equitable experiences towards competency development in a clinical placement landscape where students in the same programme can receive quite different experiences.

### Simulated-based learning experiences through telepractice

As highlighted earlier, research to date in SLP simulation has largely employed face-to-face encounters as the delivery modality. However, telepractice is a growing area of healthcare and education delivery. Telepractice is the provision of services though telecommunications networks. Through telepractice the “tyranny of distance” can be overcome, opening up services to rural and remote clients to address inequities of access, increasing demand for SLP services [39], as well as providing more flexible options in service delivery and, importantly for universities, the potential to build capacity in clinical placements. Medical and nursing student training programmes have incorporated simulation and telepractice through role-play to develop medical students’ telepractice skills [40], as well as to provide students in simulation environments access to simulation facilitators located remotely [41, 42] or provide remote students access to SPs in a learning activity [43] or examination [44].

The use of telepractice in SLP is increasing [39, 45], with systematic reviews [46, 47] demonstrating equivalent or better outcomes and advantages for telehealth over traditional clinical face-to-face service delivery, and positive stakeholder themes [48] further support the feasibility and acceptability of telepractice. Integration into university programmes to facilitate uptake and ensure graduates are confident with telepractice is paramount [49]. Indeed, Section 3.7 of Speech Pathology Australia’s Position Statement: *Telepractice in Speech Pathology* (2014) [39] states that, “The uptake and sustainability of telepractice as a model of care requires that educational programs include evidence-based theoretical and practical training of telepractice in their curriculum” (p. 6). Although telepractice has been researched, with positive outcomes, in the practice areas of speech, language, fluency, voice, swallowing, hearing disorders, and craniofacial and head and neck disorders [39], to date, just one study has investigated telehealth mentorship to SLP students in AAC modelling [38]. However, developing students’ AAC management skills through both simulation and telepractice has not been explored.

### The current study

The study was exploratory and represented a starting point for describing and evaluating the feasibility of embedding AAC training through simulation into our SLP curricula to address both a specialised practice area and as an accreditation imperative to show student competencies in this area. The simulated-based learning experience (SLE) used SPs and was a sequence of two related modules focused on providing students with AAC skills in assessment, clinical reasoning, and management. Specifically, the aims were to address the following research questions: (1) Does a simulated-based learning experience in AAC, delivered through telepractice, positively impact students’ perceptions of confidence and skill development in AAC, and (2) How do student’s perceive the delivery of AAC services through telepractice, specifically a videoconferencing format.

## Method

A prospective repeated measures cohort design was employed. All students enrolled in their first year of a 2-year Master of Speech Pathology programme at Griffith University, Australia, participated in the AAC simulation at the end of Semester 2. The simulation was as a mandatory activity to demonstrate practical competencies in the multimodal communication range of practice area in a whole-cohort and equitable manner, which is important for accreditation purposes. Participation in the associated research, however, was voluntary. The SLE reported here was delivered on two occasions, 12 months apart (i.e., 2013 and 2014), representing two student cohorts. Ethical clearance was obtained through the Griffith University Human Research Ethics Committee.

### Participants

Out of 63 students across two cohorts, 52 students provided informed consent to participate. The mean student age in cohort one (*n* = 34) was 27.9 years (SD = 7.91, range = 20 to 49 years), and 97.1% were female. The mean student in cohort two (*n* = 18) was 26.3 years (SD = 6.22, range = 20–38 years) and 96.6% were female. All participants had previously completed around 34 full-time clinical placement days (range of 30–38) and had previous simulation experience; the first cohort of students had participated in two previous simulation experiences which were both delivered via telepractice, while the second cohort of students had participated in three previous simulation experiences, two of which were delivered via telepractice. No prior simulations had addressed AAC. For both cohorts, a problem-based learning (PBL) set (PBL case, lectures, clinical skills tutorials) targeting AAC had been completed immediately prior to participation in the SLE. The SLE occurred at the end of the students’ first year of study.

### Procedure

The AAC simulation package for the SLE comprised two modules delivered over 1 day. Students worked in pairs with SPs trained to portray the role of an adult client with motor neurone disease. Module 1 (*Assessment*) required students to conduct a detailed assessment with the client, to gain a holistic understanding of their communication needs and communication profile. Module 2 (*Management*) required students to plan and deliver an intervention session with the client, culminating in the recommendation of a high-tech AAC device. Groups of four students were based in a small, group teaching room on campus for each module, with a SP and a simulation facilitator located in a separate room, also on campus. The SLE used the WebEx® videoconferencing platform, which required a computer, microphone, camera, and internet connection. At no point did the students and simulated patients meet in-person.

As indicated, the SLE was supervised remotely by an experienced simulation facilitator who was a qualified speech-language pathologist trained in simulation, and each facilitator supervised four students who were paired. Both modules consisted of a 6-step process, as follows:

1. *Pre-simulation preparation.* A resource package containing written case data, assessment information and instructions on working in pairs was provided to students for review a few days prior to the simulation.
2. *Briefing.* On the day of the simulation, students were introduced to the simulation and the environment by their remote facilitator. This included providing an outline of the simulation modules and procedures, and an introduction to telepractice and the technology.
3. *Simulation 1*. For 1 hour, the first pair of students undertook assessment of a simulated patient via telepractice, while the second student pair observed and took notes about their peers’ performance.
4. *Debriefing 1*. The remote facilitator led a 15-min debriefing session via telepractice, directing reflection and feedback from the (1) peer observers (2) student pair, (3) simulated patient, and (4) the facilitator, themselves. The advocacy enquiry approach to feedback [48] was used by the facilitator.
5. *Simulation 2*. For 1 hour, the second pair of students undertook assessment of a different simulated patient, while the first student pair observed and took notes about their peers’ performance.
6. *Debriefing 2*. This replicated *Debriefing 1*.

### Evaluation measures

Data were collected pre- and post-SLE using a range of questionnaires designed to elicit quantitative and qualitative data regarding the students’ perceptions of confidence, reaction, learning, and behaviour and specific questions targeting telepractice and system usability. To ensure anonymity, survey responses were coded using a unique code. For the first student cohort, data were collected using paper-based surveys which were electronically entered by a research assistant; for the second student cohort, data collection was via the secure web-based program, SurveyMonkey®. All surveys were completed immediately prior to commencing the SLE, and immediately after.

Specifically, the Student Confidence Questionnaire, adapted from Blackstock and colleagues’ [50] and Watson and colleagues’ [16] seminal physiotherapy RCT comparing simulation learning and face-to-face clinical experiences, was implemented pre- and post-SLE. This tool describes the extent of adult learning from the students’ perspective, according to Level 2 “learning” of Kirkpatrick’s evaluation of adult learning and is a reliable means of measuring student confidence across domains of communication, assessment, and management [16]. The tool has 24 items with 5-point Likert scale (where 1 = strongly disagree and 5 = strongly agree) in response to statements introduced by “I feel confident in my ability to ….” Its pre- and post-SLE administration was to ascribe any change in confidence to the simulation activity rather than other course work or experiences.

Immediately post-SLE, three other tools were applied, namely, (1) the Impact on Clinical Performance questionnaire (Additional file 1), (2) Ryan’s Intrinsic Motivation Inventory (IMI) [51], and (3) Brooke’s System Useability Scale (SUS) [52].

The perceived Impact on Clinical Performance questionnaire measured student perceptions of the impact of simulation on their future clinical performance across 3 domains: (i.e., communication, assessment, management), rated on a 7-point Likert scale (1 = strong negative impact, 4 = neutral impact, 7 = strong positive impact). Two open-ended statements further explored perceptions of the most and least effective aspects of the simulation that may impact on clinical performance. The statements were as follows:

1. Please describe the most effective part of the simulated learning experience that positively impacted on your clinical performance.
2. Please describe the least effective part of the simulated learning experience that not positively impacted on your clinical performance. How could this be improved?

The IMI was used to describe the experience and extent of adult learning from the student’s perspective, according to Level 1 “reaction” of Kirkpatrick’s evaluation of adult learning [51]. The IMI is a valid and reliable 39-item tool that measures student interest/enjoyment, perceived competence, value/usefulness (across communication, assessment and management), and perceived level of tension performing a given activity. Responses are rated on a 7-point Likert scale regarding truth of the statements (1 = not at all, 4 = somewhat, 7 = very true).

The SUS was used to gather student perspectives about the useability of the WebEx® system to deliver client management. This tool has been widely used in the literature to determine the useability of a product, such as a website, mobile phone, or web application and operates on the premise that useability encompasses effectiveness to complete tasks that are quality in output, efficiency of product performance, and user satisfaction with the product [52]. It is a 10-item scale with a 5-point Likert scale rating (1= strongly disagree and 5= strongly agree) and allows for a score out of 100 to be determined. A score of 70 and above is considered acceptable while between 50 and 70 is considered marginal and suggests changes are needed [53]. Four open-ended statements requesting further information about system useability were included to provide more in-depth information about users’ experiences with the system to inform future system improvements. The statements were as follows:

1. Describe any aspects of the videoconferencing system that worked well.
2. Describe any aspects of the videoconferencing system that need improvement.
3. Explain any advantages of using videoconferencing to deliver management to standardised patients.
4. Explain any limitations of using videoconferencing to deliver management to standardised patients.

One additional statement requesting information about the overall effectiveness of the SLE was included for the second cohort of students (*n* = 18). Although other evaluation measures in this study explored students’ perceived impact of the simulation on student communication skills, assessment, and management practices generally, this question specifically targeted students’ perceptions of the SLE related to their preparedness for working with adult clients with progressive neurological conditions requiring AAC. Students responded using a 5-point Likert scale (1= strongly disagree and 5= strongly agree).

### Data analyses

The data for the two student cohorts was pooled for analyses. Quantitative data were analysed descriptively using IBM SPSS version 22.0. Prior to this analysis, tests of normality were undertaken on each variable (Shapiro-Wilk test, *p* > 0.05). As most data were not normal, nonparametric measures were used. Wilcoxon signed-rank tests were used to investigate changes in students’ confidence prior to and following the simulations. Significance was set at *p* < 0.05.

Qualitative data from open-ended responses were reviewed and analysed using content analysis procedures [55]. Data were examined for keywords, phrases, and concepts which were given initial codes and grouped into subcategories by an independent research assistant. The codes and subcategories were crosschecked by a second researcher for accuracy and consistency. Any disagreements were discussed until consensus was reached. Related subcategories were discussed and combined into overarching categories. Definitions for each category were developed, and exemplar quotes were identified to illustrate each subcategory.

## Results

Of the 63 students participating in the SLE, 52 consented and responded to all pre- and post-simulation measures (82.5% response rate), except for the Impact on Clinical Performance questionnaire which had 51 respondents. First, students’ perceptions about their SLE in AAC will be presented, followed by their perceived experiences around using telepractice and the videoconferencing platform to deliver AAC services.

### Student Confidence questionnaire

There was a significant increase in student confidence from pre-SLE to post-SLE across the three collapsed domains of communication, assessment, and management (see Table 1).

**Table 1:** Student Confidence Questionnaire results pre- and post-simulation on communication, assessment, and management skills in AAC

	Pre- median (IQR)	Post- median (IQR)	Wilcoxon signed ranks	
			*Z*	*p*
Communication	3.82 (3.55–4.00)	4.00 (3.96–4.18)	− 4.731	< .001
Assessment	3.33 (3.00–3.67)	4.00 (3.67–4.00)	− 5.466	< .001
Management	3.00 (2.75–3.50)	4.00 (3.40–4.00)	− 5.611	< .001

### Impact on Clinical Performance questionnaire

Students perceived that the simulation experience had an overall positive impact on the development of their communication, assessment, and management skills (see Table 2).

**Table 2:** Student perception of the impact of simulation on communication, assessment, and management skills in AAC (*n* = 51) (1 = strong negative impact; 4 = neutral impact; 7 = strong positive impact)

	Mean (SD)	% reporting positive impact	% reporting neutral impact	% reporting negative impact
Communication	5.53 (.731)	96.1% (49)	3.9% (2)	0% (0)
Assessment	5.31 (.883)	86.2% (44)	11.8% (6)	2% (1)
Management	5.31 (.860)	84.3% (43)	15.7% (8)	0% (0)

When examining student perceptions about the most and least effective parts of the SLE, four key categories emerged: (1) receiving feedback was useful for learning, (2) providing clinical experience in the simulation learning environment was useful for learning, (3) working in groups was perceived as both beneficial and restricting; and (4) feeling underprepared for the simulation. Under category 1, students reported that receiving constructive feedback on clinical performance from the SPs, the facilitator, and other students was the most beneficial aspect of the simulation.

“I really liked the feedback from the [facilitator], client and students. It was very structured and specific.” Female, aged 36

Category 2 highlighted that students perceived the simulation as a realistic and safe environment in which general clinical skills could be tested and refined. Particularly, students appreciated the opportunity to further develop their communication skills by interacting with a SP.

“It [the simulation] provided me with experience in working with adult population which I haven't done before, assessment and intervention, specifically creating a management plan and proposals” Female, aged 49

“It felt like I was interacting with a real patient, so it was good to practice my communication skills in a realistic way.” Female, aged 35

“The simulation has helped me gain confidence, the ability to speak naturally and incorporate my knowledge in a client-centred manner in a safe and secure environment” Female, aged 37

Category 3 revealed students’ perceived working in groups as both beneficial and restrictive. They found observing others complete the simulation activity beneficial for their own learning.

“Seeing other students was great because it modelled for me strengths and weakness which I could interpret” Female, aged 23

However, working in a pair while completing the activity was perceived as restricting for some students, as it altered the way students would usually interact with a patient.

“We were conscious of each getting an 'equal share' of time and we were being assessed. So instead of elaborating and going with it as you were going … we would stick to the form and ensure we didn't touch on the other person's areas.” Female, aged 29

Within category 4, students reported feeling underprepared for the simulation and felt that aspects of the simulation structure limited their learning. Students felt more information and time, both within and prior to the simulation, were required to understand what was expected of them and to feel adequately prepared.

“Lack of information provided about the structure of the [simulation] day. Lack of time (& limited prior knowledge) to prepare for this session”. Female, aged 24

Students in the second cohort of the study (*n* = 17) were further asked to indicate their response to the statement: *The simulation education has made me better prepared to manage a client with a progressive neurological condition and make AAC recommendations*. Most of the students (94.1%) reported either strong agreement or agreement with this statement, while the other 5.9% of students reported they were unsure (Additional file 1).

### Intrinsic Motivation Inventory

Mean scores for each of the five IMI domains immediately post-SLE were interest (5.1, SD = .90); competence (4.77, SD = .82); tension (4.24, SD = 1.20); communication (5.86, SD = .89); assessment (5.18, SD = 1.26); management (5.81, SD = .91).

### Telepractice and the System Useability Scale

Using the WebEx® videoconferencing platform resulted in a mean score of 64.95 (*SD* = 11.53, range = 32.5–87.5). Twenty-three students (44.23%) rated the system 70 or above, which is the cut-off for a system to be deemed useable (Brooke, 1996). Students reported system useability issues including feeling a disconnect with patients through not having them physically present. This reportedly reduced the students’ ability to build rapport and to acknowledge, make and respond to non-verbal communication with the simulated patient:

“Maintaining eye contact and engaging with the client is more challenging” Female, aged 22

Furthermore, the disconnect limited the provision of physical resources the students felt they should provide their patients:

“We can’t interact physically to actually show them [patients] how to use devices we offer” Female, aged 28

Students acknowledged potential for technological limitations and reported that the use of telecommunication during an SLP consult may cause disruptions to patient communication. In particular, students identified weak internet connectivity, limited audio-visual equipment, and the technological receptiveness of the patient as barriers to communication:

“Sometimes if there is a weak connection it will cut out” Female, aged 23

“Patients/clients need to download the program on their computer and have basic computer literacy skills which may not be the case for all” Female, aged 27

## Discussion

While a growing body of simulation literature focused on SLP curricula is emerging, this study represents the first known use of simulation with an adult client with complex communication needs requiring AAC. The current study explored the perceptions of SLP students undertaking such a learning experience and their perceptions about using telepractice to deliver their service. Our preliminary results revealed that overall student perceptions were positive, and the majority of the students perceived the SLE, delivered via telehealth, as a valuable addition to their learning and skill development both in AAC and more broadly.

### Perceptions about the SLE in AAC

Student confidence across all domains (communication, assessment, and management) increased from pre-to post-SLE. Although statistically significant, the increases were small. It would appear that student confidence was already moderate prior to undertaking the simulation package, which may be partly attributable to the intentionally scaffolded and recent, related curricular content (PBL case in AAC and lectures) and a field trip to a facility specialising in assistive devices in the days prior to the simulation. Although small, the significant increase in confidence suggests the value of practical experience provided by this simulation as a capstone experience when added to the multimodal communication curricular content and field trip. This relatively small increase in confidence post-simulation contrasts with other studies in SLP simulation. The work of Ward and colleagues [54] in paediatric dysphagia human patient simulation found small changes in student confidence prior to and following lectures related to the simulation content, but following the hands-on simulation, these confidence changes were large. An important caveat is that Ward and colleagues [54] neither used SPs nor telepractice delivery; hence comparisons must be guarded. Nonetheless, one possibility for this discrepancy may be the role that previous clinical placement experience played on student confidence levels. Although Ward and colleagues’ [54] participants also were Master’s students in their first year of study, they had undertaken approximately 25 h of clinical practice in a university clinic (just above novice level), whereas the current student participants had undertaken 34 full-time days (255 h) of clinical placement prior to the simulation (intermediate level). Our students, therefore, were considerably more clinically experienced at the time of simulation, and it is known from the literature that greater clinical experience often translates to greater confidence [8]. The nature of the questions asked pre- and post-SLE also may have contributed to this differing finding. Ward and colleagues [54] used questions that were quite specific to paediatric practice, specifically dysphagia practice in paediatrics. In comparison, and with one exception, our questions were more generic and could be broadly applicable to a range of SLP practice areas within simulation, which may have influenced the extent and scope of reporting in student confidence pre- and post-SLE.

Pleasingly, the majority of students felt that the simulation experience had a positive impact on their performance in the areas of communication, assessment, and management, with communication yielding the strongest positive ratings. Only the assessment domain received one negative result. Perhaps more interesting is the proportion of “neutral” responses received (i.e., 3.9% for communication compared to 11.8% for assessment and 15.7% for management). It is important to note that students completed the questionnaires immediately after finishing the SLE. As such, students may not have had sufficient time to determine their perceptions, making it more difficult to appreciate the impact of the SLE during a single iteration; perhaps, if *post hoc* data had been collected through a reflective piece within 48 h, a second AAC simulation at a later stage, or interviews with students who then clinical placement where AAC skills were needed, more benefit from the current SLE might have emerged. Nonetheless, when specifically asked about managing an adult client with a progressive neurological condition and making AAC recommendations, almost all students (94.1%) reported they felt better prepared following the SLE. Regarding the strong positive ratings for the communication domain, this may have been contributed to by students having the chance to develop communication skills across the day (during both the assessment and management modules) whereas assessment skills were honed only in the first module and likewise for management skills in the second module.

On the IMI, students reported relatively high interest and enjoyment in the task (*M =* 5.10 on a 7-point scale) and placed high value/usefulness of the task on communication (*M* = 5.86) and management (*M* = 5.81) domains. However, somewhat lower levels of perceived competence (*M* = 4.78) and moderate levels of tension (*M =* 4.24 on a 7-point scale) were found.

IMI’s competence-focused questions explored concepts of self-satisfaction with task performance, self-perception of “doing well” and “being good” at the task in addition to feeling competent and skilled. The modest self-ratings in this domain are perhaps unsurprising as the students were all in their first year of a 2-year SLP programme, and the practice area and client population were new. This is a similar finding to Ward and colleagues [54] where students’ actual perceptions of their abilities remained low following the simulation experience and across time (up to 8 months post-simulation).

IMI’s tension-focused questions explored concepts of nervousness, relaxation, anxiety, and pressure related to working through the task. In contrast to the moderate levels of tension reported in the current study, Ward and colleagues [54] found low levels of student-reported anxiety related to learning skills through simulation. However, as highlighted, the Ward and colleagues’ [54] simulation was quite different in scope and focus, targeting paediatrics through human patient simulations (models) in a face-to-face environment, with tasks emphasising communication skills as well as paediatric dysphagia technical skills (e.g., patient handling). Our study targeted adults through SPs in a telepractice simulation environment with tasks focused on communication and technical skills in AAC assessment and management. Additionally, our students were aware that their competencies were being scrutinised; hence, anxiety around this is a likely contributor and came through as a subcategory. As in real-world clinical practice, different caseloads, service delivery modes, and management tasks may lead to greater or lesser feelings of tension. The current status of SLP simulation learning research is such that we do not know the individual parameters or combinations of these which are more difficult or tension-provoking for students. However, our results suggest that the combination of complex adult clients, where both assessment and management decisions need to be demonstrated and using a telehealth platform may contribute individually or collectively to greater feelings of tension. More generally, it has been suggested that student tension/anxiety in a clinical task may arise due to students feeling responsibility for clients, the presence of a new learning situation, lack of appropriate theory to meet client needs, high expectations of themselves, and the amount of preparation that is required for the practical experience [56]. Although our students felt that the simulation and SPs were real, they reported feeling underprepared with lack of information and knowledge; hence, it is plausible that the moderate levels of tension may have contributed to some of the factors identified by Chan, Carter, and McAllister [56], with preparedness being prominent.

### Perceptions about telepractice

Regarding system usability, overall, our students perceived that the videoconferencing platform needed improvement, with over half the students rating the system as not meeting the criteria for acceptable useability. However, the open-ended statement responses revealed that most technical difficulties arose from internet connectivity issues and limitations in the audio-visual capabilities of the computer hardware rather than the WebEx® software itself. In recent times, marked improvements have occurred in videoconferencing quality and ease, so connectivity should become less of an issue in the future. Students also expressed some difficulty communicating with patients via telepractice, reporting feelings of disconnectedness. Hayden and colleagues [38] reported a similar outcome where videoconferencing technology was found to be a barrier to communication between simulation facilitators and medical students. Reinets, Teuss, and Bonney [36] found that medical students participating in a telepractice role-play were observed to struggle with unfamiliar equipment and processes. In the present study, all students had previous experience using telepractice in two simulation activities; however, their most recent experience had been approximately 6 months earlier. Students may have benefitted from more specific instruction and experience in telepractice in their preparation to feel more comfortable and to develop strategies to facilitate communication across this medium. Nevertheless, that students were able to experience highly authentic telepractice consultations, which is important for the contemporary workforce, and reflect on both the benefits and challenges of this growing service delivery model, is important learning.

### Limitations and future directions

Limitations in this study present opportunities for further research in this field. First, measures in this study were perception-based; hence they neither were observable nor assessed actual skill attainment. Application of the skills acquired from the simulation to real-life clinical scenarios was not followed up, so this would be an important addition for future work in this area to capture both Level 3 “changes in performance” and Level 4 “results” of Kirkpatrick’s training and evaluation model. Triangulating data from students’ future clinical placement evaluations and their reflections may assist in identifying the broader impact of this SLE. Second, the data were collected from a small sample of Masters-level students from one institution. Masters-level students potentially have greater depth of background and life experiences than undergraduate students; generalisability of our findings to undergraduate students or other institutions cannot be assumed but is an important line of future inquiry. Further research in AAC simulation is essential to understand how different parameters influence student learning during SLEs, as well as the extent to which simulation can be used in SLP curricula and how such activities may augment real-life clinical practice. Finally, the impact of using telepractice as the service delivery mode yielded some negative student responses and this needs to be further unpacked. Future iterations of our SLE as an in-clinic simulation would provide some data for comparison with our telepractice outcomes.

## Conclusion

As an exploratory study, the overall outcomes suggest that embedding a whole-cohort SLE in AAC into a Masters-level speech pathology curriculum was feasible and had a number of positive perceived benefits for students as well as promise as evidence for satisfying multimodal communication requirements for accreditation. Specifically, we explored student perceptions of confidence, clinical skill development, and their overall learning experience, as well as their perceptions of the using a telepractice service delivery. Students perceived the activity to improve their confidence and impact positively on their clinical development in AAC across all areas explored (communication, assessment, and management). Simulation is rarely without some challenges, and students reported feeling underprepared for the SLE. However, the main challenges related to the telepractice delivery mode. Specifically, students encountered system useability issues and reported feelings of patient-SLP disconnect due to the technology. Despite these challenges, following the SLE, students reported feeling better prepared to manage a client with a progressive neurological condition and make AAC recommendations. Thus, the results from this study further affirm the value of clinical and curricular opportunities for SLP student clinical skill development using simulation and telepractice and have provided some leads for refining the current activity and future research.

## Supplementary information

**Additional file 1.** Impact on clinical performance.

## Data Availability

The authors are willing and able to provide research data on request.

## Abbreviations

AAC: Augmentative and alternative communication
SLE: Simulation learning environment
SLP: Speech-language pathology
SPs: Simulated patients
