# Supplementary material for: A simulation-based learning experience in augmentative and alternative communication using telepractice: speech pathology students’ confidence and perceptions
Source: Adv Simul (Lond). 2019 Dec 20;4(Suppl 1):23. doi: 10.1186/s41077-019-0113-x (PMC6924137; doi:10.1186/s41077-019-0113-x)
Supplement: Supplementary file 1 — Additional file 1. Impact on clinical performance. [file 41077_2019_113_MOESM1_ESM.docx]

**Additional file 1**

**Impact on clinical performance**

We are interested in your perception of the impact that the addition of simulated learning has had on your performance during this clinical placement. Please rate the level of impact by placing an X in the appropriate box for each of the following 3 statements, using the following scale:

**1 2 3 4 5 6 7**

Strong negative Neutral Strong positive

impact impact impact

**Rate your perceived impact that the addition of simulated learning has had on your:**

|  |  | **1** | **2** | **3** | **4** | **5** | **6** | **7** |
| --- | --- | --- | --- | --- | --- | --- | --- | --- |
| 1. | Performance in the area of **communication** |  |  |  |  |  |  |  |
| 2. | Performance in the area of client **assessments** |  |  |  |  |  |  |  |
| 3. | Performance in the area of client **management** |  |  |  |  |  |  |  |

Please describe the most effective part of the simulated learning experience that positively impacted on your clinical performance during this clinical placement

Please describe the least effective part of the simulated learning experience that did not positively impact on your clinical performance during this clinical placement. How could this be improved?
